# Comparative outcomes of cemented versus cementless stems in bipolar hemiarthroplasty for femoral neck fractures

**DOI:** 10.1097/MD.0000000000039946

**Published:** 2024-10-11

**Authors:** Tomoya Ono, Nobuyuki Watanabe, Kazuo Hayakawa, Shingo Kainuma, Hiroki Yamada, Yuya Waseda, Yoshihiro Kanda, Muneyoshi Fukuoka, Haruhiko Tokuda, Hideki Murakami, Gen Kuroyanagi

**Affiliations:** a Department of Orthopedic Surgery, Tosei General Hospital, Seto, Japan; b Department of Orthopedic Surgery, Nagoya City University Graduate School of Medical Sciences, Nagoya, Japan; c Department of Metabolic Research, Research Institute, National Center for Geriatrics and Gerontology, Obu, Japan; d Department of Clinical Laboratory, National Center for Geriatrics and Gerontology, Obu, Japan; e Department of Rehabilitation Medicine, Nagoya City University Graduate School of Medical Sciences, Nagoya, Japan.

**Keywords:** cemented fixation, cementless fixation, femoral neck fracture, hemiarthroplasty

## Abstract

We aimed to compare the clinical and surgical outcomes of cemented vs uncemented bipolar hemiarthroplasty in the treatment of femoral neck fractures in the elderly. Patients (n = 99) without preoperative cardiopulmonary problems undergoing bipolar hemiarthroplasty for femoral neck fracture between August 2015 and February 2019 were randomly divided into cemented (group C) and uncemented (group U) stem fixation groups. Mean operative time, mean intraoperative blood loss, and percentage of intraoperative use of vasopressors, pre- and postoperative activities of daily living (ADL), incidence of postoperative complications, and radiological evaluation of stem alignment were evaluated. A total of 99 patients were included (group C, n = 42; group U, n = 57). Group C had a significantly longer mean operative time (*P* < .001) and a significantly higher percentage of intraoperative vasopressor use as compared to group U (*P* < .05). In contrast, the amount of intraoperative blood loss was similar between the 2 groups (*P* = .30). Likewise, there was no statistically significant difference in pre- and postoperative ADL performance between the groups (*P* = .70 and .44, respectively). Postoperative computed tomography revealed that stem anteversion was higher in group C than in group U (*P* < .05). Cemented and uncemented stems were equivalent in terms of blood loss and postoperative complications in patients with femoral neck fractures. Uncemented stem showed advantages in reducing operative time and intraoperative vasopressor administration. Also, fixation method was not investigated in this study.

## 1. Introduction

Femoral neck fractures are 1 of the most common fractures in elderly individuals with osteoporosis. In 1990, the reported incidence of hip fractures, including femoral neck fractures, was 1.26 million; it is projected to reach up to 2.6 million by 2025 and 4.5 million by 2050.^[1]^ The treatment of femoral neck fractures is typically surgical, involving procedures such as osteosynthesis or hip hemiarthroplasty; in the case of the latter, bipolar hemiarthroplasty is more widely used. Total hip arthroplasty is also an option for treatment of femoral neck fractures.^[2]^

In bipolar hemiarthroplasty, there are 2 options for stem fixation – cemented and cementless stems. Cemented stems are often preferred and show good clinical outcomes in elderly patients with osteoporosis and wide medullary canals.^[3–5]^ Cemented stems also have the advantage of reducing the risk of periprosthetic fractures.^[6]^ However, the use of bone cement is associated with complications such as intraoperative hypotension.^[7]^ In contrast, cementless stems are associated with a higher incidence of intraoperative or postoperative fractures.^[8]^ However, it remains unclear whether cemented or cementless fixation affects intraoperative blood loss and the intraoperative use of vasopressors in the treatment of femoral neck fractures in elderly patients.

The purpose of this study was to compare cemented and cementless stem fixation in hemiarthroplasty for elderly patients with femoral neck fractures in terms of operative time, intraoperative blood loss, and intraoperative use of vasopressors.

## 2. Methods

### 2.1. Study patients

**Figure 1.**
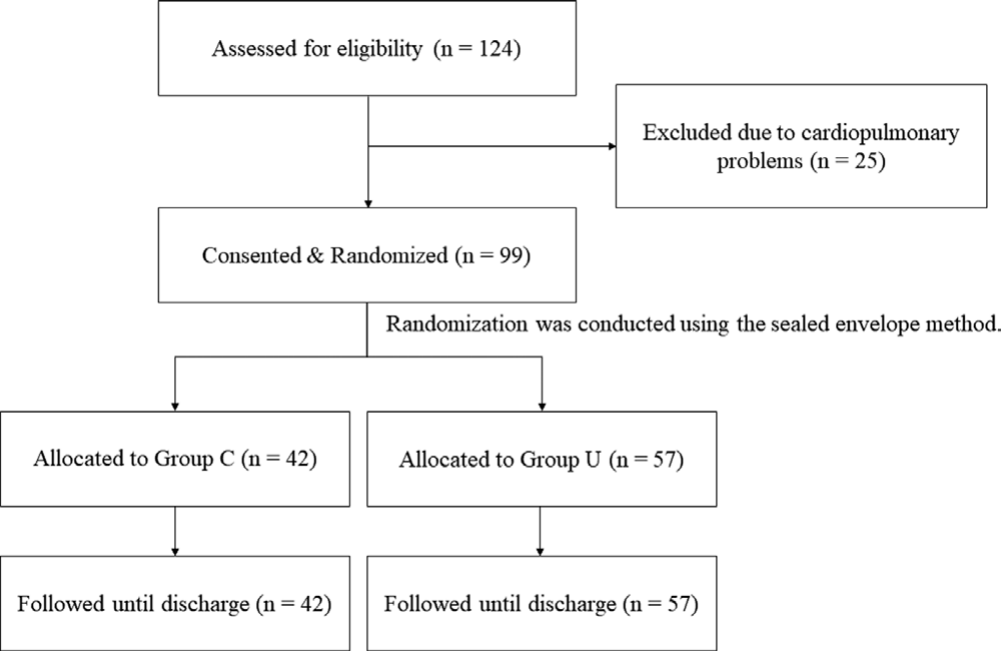
Flowchart of participant selection through the trial.

This prospective cohort study included 124 patients who underwent bipolar hemiarthroplasty at a trauma center between August 2015 and February 2019 (Fig. 1). Patients with preoperative cardiopulmonary problems were excluded. Thus, 99 patients were finally included in this study. The study was approved by the local Research Ethics Committee (approval no.: 465). Written informed consent was obtained from all patients before enrollment. This manuscript adheres to the applicable STROBE checklist (Supplemental Digital Content, http://links.lww.com/MD/N697).

The included patients were randomly assigned to undergo bipolar hemiarthroplasty using either cemented (group C; n = 42) or uncemented stem fixation (group U; n = 57) by an envelope method. All surgeries were performed by the same surgical team using a posterior approach under spiral anesthesia. In group C, the Exeter (Stryker Co., Kalamazoo, MI, USA) with a polished double taper design was used, whereas the Accolade II (Stryker Co.) with a tapered wedge design was used in group U. A third-generation cementation technique using a cementing gun and pusher was consistently applied to all patients in group C. Vasopressors were administered in both groups when systolic blood pressure dropped below 80 mm Hg during surgery.

### 2.2. Outcome measures

The mean values for the operative time, intraoperative blood loss, and percentage of intraoperative use of vasopressors during surgery were assessed for all patients. The perioperative hemodynamic loss of change was calculated using Nadler formula^[9]^ based on hemoglobin levels before and 7 days after surgery. The performance of ADLs was assessed preoperatively and at discharge as previously described.^[10]^ Additionally, the incidence of perioperative complications, including deep vein thrombosis (DVT), was investigated.

For radiological evaluation, postoperative stem alignment was examined by computed tomography (CT). The stem anteversion was measured as the angle between the femoral neck axis and the posterior condylar axis of the femur on axial CT images using the method described by Pierchon et al.^[11]^ Additionally, the stem valgus angle, femoral offset, and leg length were measured on frontal radiographs.^[12,13]^ Bone density of the femoral neck was classified according to the Singh index, which is based on the trabecular pattern in the femoral head and neck visualized on radiographs after surgery, and graded from 1 to 6 depending on the disappearance of the normal trabecular pattern.^[14]^ Grades 1 to 3 indicated osteoporosis, whereas grades 4 to 6 were considered not to indicate osteoporosis. The shape of the femoral bone marrow cavity was evaluated using the canal flare index (CFI).^[15]^ Lastly, implant survival at hospital discharge was also evaluated.

### 2.3. Statistical analysis

All data were analyzed using SPSS (version 25.0, IBM Corporation, Armonk, NY). Continuous data were expressed as mean ± standard deviation (SD). The Mann–Whitney *U* test was used to compare the 2 groups for continuous variables. The chi-square test was used to compare categorical variables including sex, intraoperative vasopressor administration, complications, postoperative stem alignment, Singh index, and implant survival until discharge from the hospital. A *P*-value of < .05 was considered statistically significant.

## 3. Results

The total number of participants in this study was 99 (male: n = 15; female: n = 84), with an average age of 80.6 ± 7.7 years. The average age of the patients was 83.3 ± 6.2 years in group C and 78.7 ± 8.1 years in group U; patients in group C were significantly older than those in group U (*P* < .05) (Table 1). There were no statistically significant differences between the 2 groups regarding the patients’ sex (*P* = .62), body mass index (BMI) (*P* = .41), waiting time for surgery (*P* = .89), or average length of hospital stay (*P* = .41).

**Table 1:** Demographic and clinical data of the study patients.

Variables	Group C (n = 42)	Group U (n = 57)	*P*-value
Age (yr), mean (SD)	83.3 (6.2)	78.7 (8.1)	<.05
Sex, n (%)			
Male	5 (11.9)	10 (17.5)	.62
Female	37 (88.1)	47 (82.5)	.62
Height (cm), mean (SD)	149.3 (7.3)	152.5 (8.2)	.06
Body weight (kg), mean (SD)	47.7 (9.3)	47.6 (7.9)	.77
Body mass index (kg/m^2^), mean (SD)	21.4 (3.7)	20.5 (2.8)	.41
Preoperative waiting period (d), mean (SD)	5.2 (8.0)	3.6 (2.7)	.89
Hospitalization duration (d), mean (SD)	27.6 (10.8)	29.6 (13.1)	.41

SD = standard deviation.

Group C indicates cemented stem fixation and group U indicates cementless stem fixation.

The mean operative time in group C was significantly longer (71.5 ± 11.7 minutes) than in group U (58.5 ± 16.6 minutes) (*P* < .001) (Table 2). The average intraoperative blood loss was similar between the groups (group C: 191.7 ± 174.4 mL; group U: 156.7 ± 138.6 mL) (*P* = .30). Intraoperative vasopressors were more frequently used in group C than in group U (n = 19 [45.2%] vs n = 15 [26.3%]) (*P* < .05). Perioperative hemodynamic losses, measured as the difference between preoperative and 7-day postoperative hemoglobin levels, were similar between them (group C: 289.7 ± 123.5 mL; group U: 302.3 ± 149.6 mL) (*P* = .93).

**Table 2:** A comparison of intraoperative and postoperative variables between the cemented and uncemented groups.

Variables	Group C (n = 42)	Group U (n = 57)	*P*-value
Operative time (min), mean (SD)	71.5 (11.7)	58.5 (16.6)	<.001
Intraoperative blood loss (ml), mean (SD)	191.7 (174.4)	156.7 (138.6)	.30
Intraoperative vasopressor administration, n (%)	19 (45.2)	15 (26.3)	<.05
Perioperative hemodynamic loss (ml), mean (SD)	289.7 (123.5)	302 (149.6)	.93
ADL score*, mean (SD)			
Pre-injury ADL	7.3 (3.5)	7.1 (3.8)	.70
ADL at discharge	6.3 (3.2)	6.6 (3.7)	.44
Complications, n (%)	5 (11.9)	16 (28.1)	.08
Deep vein thrombosis	4 (9.5)	7 (12.3)	.91
Periprosthetic fracture	0 (0)	1 (1.8)	1
Others	1 (2.4)	8 (14.0)	.10
Femoral anteversion (°), mean (SD)			
Preoperative femoral anteversion	17.5 (9.4)	19.1 (10.7)	.43
Postoperative stem anteversion	25.5 (9.6)	30.2 (11.3)	<.05
Postoperative stem alignment, n (%)			
Neutral	39 (92.8)	56 (98.2)	.24
Varus	2 (4.8)	0 (0)	
Valgus	1 (2.4)	1 (1.8)	
Postoperative stem offset (mm), mean (SD)	−2.1 (2.3)	−1.8 (1.9)	.49
Postoperative leg length difference (mm), mean (SD)	−3.1 (6.1)	−1.1 (5.4)	.09
Singh index, n (%)			
Grade 1,2,3	30 (71.4)	36 (63.2)	.51
Grade 4,5,6	12 (28.6)	21 (36.8)	
Canal flare index, mean (SD)	3.4 (4.0)	4.5 (4.2)	.18
Implant survival until discharge from hospital, n (%)	42 (100)	56 (98.2)	1

ADL = activities of daily living, SD = standard deviation.

* ADL score is based on the Fujibayashi classification.^[10]^ Group C indicates cemented stem fixation, and group U indicates cementless stem fixation.

There were no significant differences between the groups regarding ADL scores pre-injury (*P* = .70) and at discharge (*P* = .44). Perioperative complications were observed in 5 cases (11.9%) in group C and 16 cases (28.1%) in group U. Among these, DVT occurred in 4 cases (9.5%) in group C and 7 cases (12.3%) in group U. However, there were no statistically significant differences in the incidence of perioperative complications (*P* = .08) and DVT (*P* = .91) between the groups. In 1 patient in group U, a periprosthetic fracture was not detected on the postoperative radiograph but was detected on a CT scan on the 3 days after surgery. This patient was diagnosed with an intraoperative occult fracture and underwent reoperation.

In terms of radiographic characteristics, there was no significant difference between the groups regarding the preoperative femoral anteversion angle (group C: 17.5 ± 9.4°; group U: 19.1 ± 10.7°) (*P* = .43). In contrast, the postoperative anteversion angle was significantly higher in group U than in group C (group C: 25.5 ± 9.6°; group U: 30.2 ± 11.3°) (*P* < .05). Postoperative radiographs revealed that stem varus was observed in 2 cases (4.8%) in group C, whereas none of the group U patients had this. Stem valgus was observed in 1 patient in each group (group C: 2.4%; group U: 1.8%). The mean femoral offset compared with the contralateral side was − 2.1 ± 2.3 mm in group C and − 1.8 ± 1.9 mm in group U. The mean leg length difference was − 3.1 ± 6.1 mm in group C and − 1.1 ± 5.4 mm in group U. No statistically significant differences were observed between the groups in terms of stem positioning (varus or valgus) (*P* = .24), femoral offset (*P* = .49), and leg length discrepancy (*P* = .09).

Regarding the prevalence of osteoporosis, 30 cases (71.4%) in group C and 36 cases (63.2%) in group U presented with osteoporosis; however, this difference was not statistically significant (*P* = .51). The average CFI was 3.4 ± 4.0 in group C and 4.5 ± 4.2 in group U, with no statistically significant difference between the groups (*P* = .18).

Finally, both groups were comparable regarding implant survival until discharge from the hospital (group C: 100%, n = 42; group U: 98.2%, n = 56).

## 4. Discussion

In the present study, the cemented stem fixation group exhibited longer surgical times and more frequent use of vasopressors compared to the uncemented fixation group. The efficacy of hemiarthroplasty for treating femoral neck fractures in the elderly is widely recognized; however, the choice between cemented and uncemented stem fixation remains debatable. Although the American Academy of Orthopaedic Surgeons recommends cemented stems,^[6]^ cementation is often associated with a risk of early postoperative thromboembolic cardiovascular events, such as DVT and pulmonary embolism.^[16,17]^ Additionally, cemented fixation is related to prolonged operation times, increased blood loss, and higher local complications rates.^[18,19]^ Conversely, cementless fixation carries a risk of intraoperative periprosthetic fractures, and postoperative thigh pain. However, it offers equivalent postoperative clinical outcomes and similar risks of systemic complications and revision surgery as cemented fixation. So far, no study has compared the 2 fixation methods for bipolar hemiarthroplasty in femoral neck fractures regarding other surgical variables, such as intraoperative and perioperative blood loss and frequency of use of vasopressors. Thus, our findings in this study may add new perspectives to the treatment of femoral neck fractures in the elderly.

The relatively longer operative time observed in the cemented group in the present study may be attributed to the preparation time for setting the cement and the waiting time until the cement hardens. Some studies have also shown that the operative time was significantly longer in the cemented group than in the uncemented group,^[20,21]^ while another study found no significant difference between the 2 groups.^[22]^ Extended surgical time can lead to increase intraoperative blood loss. Movrin et al reported that the cemented group experienced increased intraoperative blood loss, early postoperative mortality, and longer surgical times.^[7]^ Although the present study observed no significant difference in intraoperative and perioperative circulating blood loss, ensuring appropriate hemostasis during surgery remains crucial for procedural safety.

Surgeons should be particularly cautious about intraoperative hypotension during cementing fixation, as it might occur due to bone cement embolism. Bone cement can decrease stroke volume and cardiac output after hemiarthroplasty for displaced femoral neck fractures.^[23]^ Olsen et al reported that bone cement usage in hemiarthroplasty for femoral neck fractures increased the incidence of intraoperative hypoxia and hypotension, identifying it as an independent risk factor for 1-year postoperative mortality.^[24]^ In our study, vasopressor use was significantly more frequent in group C than in group U, which may be attributed to temporary hypotension occurring during the cementing procedure. To the best of our knowledge, there are no reports comparing the frequency of vasopressor use during surgery for femoral neck fractures. Therefore, surgeons must consider the risk of hypotension during cementing procedure for elderly patients, and should opt for cementless fixation in cases with preexisting diseases, such as cardiovascular abnormalities, renal impairment, and anemia. Additionally, bone cement implantation syndrome (BCIS) is a potentially fatal perioperative complication associated with the cementing procedure, resulting in emergent circulatory impairment.^[25]^ BCIS is characterized by hypotension, hypoxia, and cardiac arrest, and occurs during cementing, prosthesis insertion, or immediately after surgery.^[26]^ In the present study, increased intraoperative use of vasopressors was observed in the cemented bipolar hemiarthroplasty group. Therefore, BCIS could be 1 possible explanation for the significantly higher use of vasopressors in the cemented bipolar hemiarthroplasty group. On the other hand, a systematic review analyzing the outcomes of various fixation methods for patients with femoral neck fractures reported that cemented hemiarthroplasty is widely performed.^[27]^ Additionally, a meta-analysis comparing different treatments for intra-articular displaced femoral neck fractures in elderly patients showed that cemented bipolar hemiarthroplasty presents better outcomes than uncemented bipolar hemiarthroplasty in terms of reoperation rates.^[28]^ Taken these finding into account, when performing cemented bipolar hemiarthroplasty in elderly patients with femoral neck fractures, careful monitoring and management of BCIS is important.

In terms of postoperative complications, the existing literature offers variable findings regarding the use of cemented vs uncemented fixation methods for treating femoral neck fractures. Cement infiltration into blood vessels may lead to DVT^[29,30]^; however, a previous meta-analysis and systematic review found no significant difference in the incidence of DVT between cemented and uncemented methods.^[31,32]^ Similarly, the present study found no significant difference in the incidence of DVT between the 2 groups. Regarding reoperation, a significantly higher number of revision surgeries have been reported with cemented implants, raising concerns about their long-term durability.^[33,34]^ In contrast, Scanelli et al showed that cemented stems are less prone to periprosthetic fractures than uncemented stems when used with proper fixation and cementation techniques, especially in femurs with poor bone quality.^[35]^ In the present study, a periprosthetic fracture occurred in 1 patient within the uncemented group, which could be attributed to the poor quality and thickness of the cortical bone. Therefore, it is essential to consider the shape of the patient’s medullary canal when deciding on the use of bone cement to prevent periprosthetic fractures. In addition, operative time, intraoperative hypotension, DVT, and implant-related complications are important factors that must be taken into account when making treatment choices in patients with femoral neck fractures.

This study had several limitations. First, we did not perform a preoperative power analysis to determine the sample size required to compare small differences between the 2 groups. However, a post hoc power analysis with an *α* level of 0.05 was conducted using G*Power (version 3.1.9, Heinrich Heine University, Düsseldorf, Germany). The analysis revealed that the power (1-*β*) for detecting differences in operative time and vasopressor administration rate was 0.99 for both, suggesting that the sample size was sufficient to detect these differences. Second, a significant age difference was observed between patients included in the 2 groups. Although group allocation of the study sample was done randomly, the age disparity may have influenced the outcomes. Third, patients with cardiovascular issues were excluded from this study. It is noteworthy, however, that even patients without cardiovascular problems may experience intraoperative hypotension during cemented fixation surgeries. Finally, the follow-up period was relatively short, with the average hospital stay of 28 days, including rehabilitation. Consequently, medium- to long-term outcomes after discharge were not evaluated.

## 5. Conclusion

Cemented and uncemented stems were equivalent in terms of blood loss and postoperative complications in patients with femoral neck fractures. Uncemented stem showed advantages in reducing operative time and intraoperative vasopressor administration. Also, fixation method was not investigated in this study. This study provides new insights into the surgical considerations for bipolar hemiarthroplasty for femoral neck fractures in elderly patients using cemented or cementless fixation.

## Author contributions

**Conceptualization:** Tomoya Ono, Nobuyuki Watanabe.

**Data curation:** Tomoya Ono, Nobuyuki Watanabe, Kazuo Hayakawa, Shingo Kainuma, Hiroki Yamada, Yuya Waseda, Yoshihiro Kanda, Muneyoshi Fukuoka, Hideki Murakami, Gen Kuroyanagi.

**Formal analysis:** Tomoya Ono, Nobuyuki Watanabe, Gen Kuroyanagi.

**Investigation:** Haruhiko Tokuda.

**Writing – original draft:** Tomoya Ono, Nobuyuki Watanabe, Gen Kuroyanagi.

**Writing – review & editing:** Kazuo Hayakawa, Shingo Kainuma, Hiroki Yamada, Yuya Waseda, Yoshihiro Kanda, Muneyoshi Fukuoka, Haruhiko Tokuda, Hideki Murakami.

## Supplementary Material


